# Draft Genome Sequence of *Stenotrophomonas maltophilia* SBo1 Isolated from *Bactrocera oleae*

**DOI:** 10.1128/genomeA.00905-16

**Published:** 2016-09-22

**Authors:** Frances Blow, John Vontas, Alistair C. Darby

**Affiliations:** a Institute of Integrative Biology, University of Liverpool, Liverpool, Merseyside, United Kingdom; b Institute of Molecular Biology a Biotechnology, Foundation for Research & Technology Hellas, Heraklio, Crete, Greece; c Department of Crop Science, Agricultural University of Athens, Athens, Greece

## Abstract

Bacteria of the genus *Stenotrophomonas* are ubiquitous in the environment and are increasingly associated with insects. *Stenotrophomonas maltophilia* SBo1 was cultured from the gut of *Bactrocera oleae*. The draft genome sequence presented here will inform future investigations into the nature of the interaction between insects and their microbiota.

## GENOME ANNOUNCEMENT

Members of the genus *Stenotrophomonas* reside in a broad range of environments and are commonly identified as multidrug-resistant opportunistic pathogens of humans (1). However, they are most frequently found in soils or in association with plants (2), where they are able to form symbiotic interactions (3). *Stenotrophomonas* spp. are also associated with multiple insect species (4–8), including three members of the *Bactrocera*, *B. cucurbitae*, *B. tau*, and *B. zonata* (9, 10). Due to a lack of phenotypic data, the nature of these interactions is not understood. However, the draft genome sequence of *Stenotrophomonas maltophilia* SBo1 presented here will aid future investigations into the mechanisms that underlie insect-microbe interactions and will contribute to a community-level examination of the *B. oleae* microbiota (11).

*S. maltophilia* SBo1 was cultured from the homogenate of 10 dissected guts from surface-sterilized adult *B. oleae*. Guts were homogenized in Schneider’s insect medium supplemented with 10% fetal bovine serum and spread onto brain heart infusion (BHI) agar plates. Plates were incubated at 25°C for 48 h and individual colonies were subsequently streaked on to BHI plates and again incubated at 25°C for 48 h. DNA was isolated from single colonies by boiling at 95°C for 5 min and used as the template for PCR of the 16S rRNA gene with the primers A16SF (5′-AGAGTTTGATCMTGGCTCAG-3′) and B16SR (5′-CCCCTACGGTTACCTTGTTACGAC-3′). Sanger sequencing was performed on the resulting fragment to identify the genus of bacterium as *Stenotrophomonas*. Single colonies were inoculated in to BHI broth and incubated at 25°C for 48 h and genomic DNA was extracted using the Zymo Quick DNA universal kit (Zymo) following the manufacturer’s instructions for biological fluids and cells. The following amendments to the protocol were employed: samples were incubated with proteinase K at 55°C for 30 min rather than 10 min and were eluted twice in a volume of 40 µl to give a total of 80 µl per sample. Library preparation was performed with the NEBNext Ultra DNA library preparation kit (New England Biolabs) following the manufacturers’ instructions, and sequencing was performed on an Illumina MiSeq sequencer at the Centre for Genomic Research, University of Liverpool, with paired-end 250-bp reads.

The resulting 2,512,754 reads were assembled with SPAdes version 3.7.1 (12). SPAdes generated a 4.8-Mb assembly comprising 23 contigs with an *N*_50_ of 466,519 and an average GC content of 66.1%. Genes were annotated using PROKKA version 1.5.2 (13), which produced a total of 4330 protein coding and 92 RNA genes.

### Accession number(s).

This whole-genome shotgun project has been deposited at DDBJ/ENA/GenBank under the accession number MANU00000000. The version described in this paper is version MANU01000000.
